# Does anchoring vaginal mesh increase the potential for correcting stress incontinence?

**DOI:** 10.1186/s12894-018-0363-2

**Published:** 2018-05-31

**Authors:** Zoltán Fekete, Szilvia Kőrösi, László Pajor, Zoltán Bajory, Gábor Németh, Zoltan Kozinszky

**Affiliations:** 1 Department of Urology, University of Szeged, Hungary, Semmelweis u. 1, Szeged, H-6725 Hungary; 2 Department of Obstetrics and Gynaecology, University of Szeged, Hungary, Semmelweis u. 1, Szeged, H-6725 Hungary; 3 Department of Obstetrics and Gynaecology, Blekinge Hospital, Karlskrona, Sweden

**Keywords:** Modified transvaginal mesh, Transobturator tape, Anterior colporrhaphy, Complications, SUI with POP–Q II, Clavien–Dindo classification

## Abstract

**Background:**

This study aims to explore the feasibility of anchoring a four-arm transvaginal mesh (TVM) to the mid-*urethra* to correct an anterior compartment POP–Quantification stage II–III (Q II–III) and concomitant genuine SUI.

**Methods:**

We analysed clinical data from 248 patients with stage II–III anterior prolapse and concomitant SUI who had undergone surgery at a tertiary referral centre in Hungary between January 2008 and June 2010. One hundred and twenty-four women treated with anterior *colporrhaphy* and 62 patients implanted with a conventional permanent TVM were selected as historical matched controls. Sixty-two patients received a modified permanent TVM, where the mesh was fixed to the mid*-urethra* with two stitches for the purpose of potentially correcting SUI. Surgical complications were classified using the Clavien–Dindo (CD) classification system.

**Results:**

The anti-SUI efficacy was minimally higher in the mTVM group than in the original TVM group (*p* = 0.44, 96.8% vs 91.9%, respectively), while prosthesis surgery was more effective than anterior *colporrhaphy* in improving the anterior compartment POP–Q status (96.8, 90.3% vs 64.5%, respectively). Anchoring the mesh did not increase the extrusion rate (*p* = 0.11). The de novo urge symptoms were not more prevalent among those who had received additional periurethral stitches (*p* = 1.00, 11.3% vs 12.9%). The incidence of reoperation observed in the mTVM group was non-significantly lower than that in the TVM group (*p* = 0.15, 6.5% vs 16.1%); however, the difference did not reach the level of significance. The early postoperative complication profile was more favourable among the mTVM patients (classified as CD I: 8.1%; CD II: 1.6%; and CD IIIb: 1.6%) as compared to the TVM group (*p* = 0.013).

**Conclusions:**

The new, modified mesh surgery represents an effective procedure for prolapse and concomitant SUI with a decreased risk of short- and long-term complications.

## Background

Coexisting stress urinary incontinence (SUI) and pelvic organ prolapse (POP) are highly prevalent (63–80%) [1], and the cumulative risk of surgery for POP or SUI by the age of 80 years has been estimated at 11.1% [2, 3]. Despite the availability of a wide variety of prolapse surgery, there is no consensus on the optimal treatment. Vaginal prosthetic surgery has been proposed for anterior compartment POP–Quantification (Q) stage II–III (International Continence Society/International Urogynecological Association) [4] in view of the low recurrence rate (6.7–24%) [5–8] relative to that after classical anterior *colporrhaphy* (30–40%) [6, 8, 9]. A recent Cochrane review confirms the superiority of repairing prolapse of the mid-anterior vaginal wall with permanent mesh over native tissue repair [8]. However, a worldwide spread of a range of vaginal mesh operations has resulted in a huge number of complications (e.g. infection, extrusion and de novo urge symptoms). As a consequence, the US Food and Drug Administration has published warnings with the aim of restricting the utilization of vaginal prosthesis to centres with specially trained surgeons after patients have been thoroughly informed [10]. Furthermore, it is assumed that a combination of a synthetic mesh with the sling operation [11–13] will substantially increase the cure rate of concomitant SUI. In contrast, the combined techniques represent an increased complication rate and elevated financial burden.

It is well known that the supporting position of the prosthesis differs slightly with the transvaginal mesh (TVM) and transobturator tape (TVT-O). The TVT-O sling is located under the mid-*urethra*, whereas the TVM elevates the whole anterior and middle compartment [14]. TVM produces a significantly weaker anti-SUI effect compared to that of the TVT-O since TVM elevates the urinary bladder neck [7]. Furthermore, we hypothesise that the original TVM operation can be followed by residual SUI as the strengthening of the back arms may result in a backward sliding of the entire mesh, leading to a lack of suspension of the *urethra.*

We propose a modification of the four-arm TVM procedure in an attempt to achieve a better SUI correction, without any decrease in prolapse repair efficacy. We have developed a new method where the mesh is sutured to the mid-*urethra* in order to prevent the backward sliding, and, in this way, the anterior arms mainly elevate the middle part of the urethra, potentially leading to effective anti-incontinence. In other words, the anchored mesh takes over the function of the *pubourethral ligament*, which should normally stabilize the *urethra*, but is loose in SUI.

We demonstrate an alternative operative method for POP–Q II–III repair and anti-incontinence with the TVM fixed to the mid-*urethra*. The efficacy and short- and long-term complication profiles of this new surgical technique were compared with those of historical controls involving original TVM and anterior *colporrhaphy* operations at our departments.

## Methods

The prospective longitudinal cohort study comprised 62 women who presented for the correction of SUI in conjunction with symptomatic anterior compartment POP–Q II–III at the Departments of Urology and Obstetrics and Gynaecology, University of Szeged, Hungary, between January 2010 and June 2010. The trial was conducted in accordance with the IDEAL consensus. After the idea (IDEAL stage 1) of concomitant surgery for SUI and POP was developed, ten operations were performed with a high cure rate of both symptoms in all cases after four weeks (IDEAL stage 2). Based on these data, TVM or *anterior colporrhaphy* and delayed TVT-O for SUI and coexisting POP repair have been replaced by the new surgery in our departments (IDEAL stage 3) [15]. The patients were monitored during monthly outpatient appointments in the first six months; thereafter, check-ups occurred every half year. The Institutional Review Board regularly reviewed the clinical data on the patients.

Our work was carried out in accordance with the Code of Ethics of the World Medical Association (Declaration of Helsinki) for experiments involving humans and was approved by the Ethics Committee, Faculty of Medicine, University of Szeged (Protocol No. 194/2010). The Institutional Review Board supervised patient monitoring. Written informed consent was obtained from all participants after the advantages and possible complications of the modified surgery were explained in detail.

All the patients who exhibited coexisting symptoms (both SUI and POP–Q II–III) were recruited prospectively into the study. The symptomatic POP–Q stage II–III anterior prolapse is defined as the maximum extent of the prolapsed anterior vaginal wall being within 1 cm above and 6 cm below the hymen [4, 16]. According to the international POP guidelines (the EBU and NICE guidelines) [17, 18], if the condition disrupts the patient’s life and nonsurgical treatment options have not helped, it should be treated surgically. POP repair was considered effective if a significant (> 1 cm) improvement was measured at points Aa, Ba and C and in total vaginal length (TVL) during the follow-up in accordance with the POP–Q system (International Continence Society) [4, 16]. Anti-incontinence efficacy was determined if no further SUI was diagnosed by urodynamic examination.

In all cases, urodynamic examinations comprising uroflowmetry, cystometrography and abdominal leak point pressure tests were performed before surgery to objectively determine the coexisting symptomatic SUI based on the international guidelines (the EBU and NICE guidelines) [15, 16]. The abdominal leak point pressure test was used as a standardised examination method for the evaluation of SUI with urine leakage as a sign. If the intraabdominal pressure recorded at the point of urine leakage was less than 40 cmH_2_O, the origin of the SUI was set as intrinsic *sphincter* deficiency (ISD) [19]. In the case of ISD, preoperative pelvic floor muscle training (PFMT) was recommended. If the patient was unwilling to do PFMT or the training was unsuccessful, a modified TVM operation was recommended. This was also recommended in the case of suspected urethral hypermobility – i.e. if the intraabdominal pressure at the point of urine leakage was higher than 60 cmH_2_O. Preoperative irritating voiding symptoms were not an exclusion criterion before the study.

All the data were collected prospectively. Further inclusion criteria in the study were negative urinary culturing and ineffective non-surgical treatment or patient’s unwillingness to receive conservative treatment. The exclusion criteria were as follows: occult SUI, a history of mesh use or anti-incontinence pelvic procedures, antidepressant therapy, pregnancy, whether the patient was in the puerperal period and up to six months post-partum, and cancer of the pelvic organs.

There are no clear international recommendations on surgical anterior compartment POP–Q II repair, but an increasing body of evidence suggests that TVM is a better treatment option for anterior wall prolapse ≥ stage II than traditional anterior *colporrhaphy* [7, 20, 21]. We have modified the transobturator TVM operation reported by Sergent et al. [7] by inserting two additional stabilizing sutures which fix the anterior edge of the mesh to the paraurethral tissues at the level of the mid-*urethra.* All operations were carried out with 100% polypropylene monofilament permanent meshes produced by Aspide® SURGIMESH® PROLAPSE (Aspide Médical, La Talaudière, France). The implanted vaginal prosthesis has pores which are 1.6 × 1.7 mm in size and is approved for anterior vaginal repair.

Surgery was performed under general anaesthesia with the patient in the lithotomy position with an indwelling urinary catheter. The anterior vaginal wall was incised longitudinally throughout its thickness from the cervix to 1 cm below the urethral meatus, under the mid*-urethra*. The thickness of the dissection, the location of the vaginal incision, the placement of the mesh and the closure of the incision varied only minimally, and the length of the incision varied only between 6 and 7 cm. Before insertion, all sterile meshes were soaked in iodic fluid (Betadine®). The Surgimesh® device was introduced beneath the dissection. Its four arms were then passed through the obturator membrane. The posterior part of the mesh was anchored to the anterior side of the cervix using two Prolene® 2–0 sutures (Ethicon, Issy-les-Moulineaux, France).

**Fig. 1:**
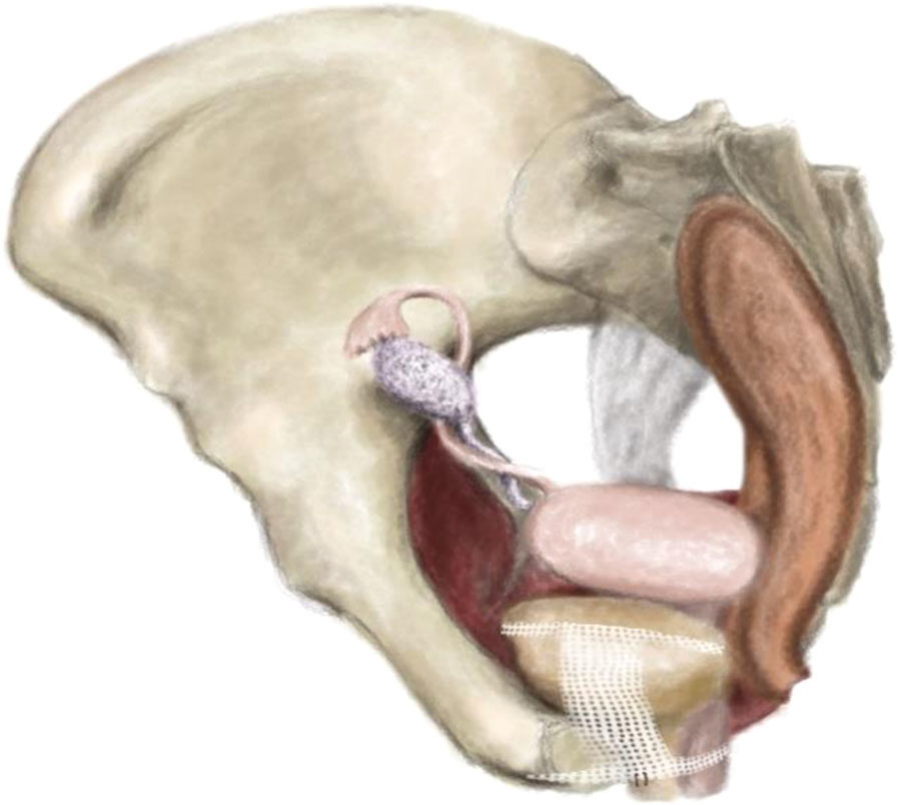
Anchoring stitches stabilize the mesh position under the mid-urethra

The mesh was then spread by securing its anterior parts beneath the mid*-urethra* using two Vicryl 2–0® absorbable sutures (Ethicon, Issy-les-Moulineaux, France) and promoting the proper elevation and closure of the *urethra* (Fig. 1). The mesh was then adjusted in a tension-free manner beneath the distal part of the urethra and bladder, and the anterior vaginal wall was closed using Monocryl® 3–0 absorbable sutures (Ethicon, Issy-les-Moulineaux, France) with a slight colpectomy. All the operations were performed by the same two experienced senior surgeons, and there were no differences in the operative processes.

Prophylactic preoperative antibiotics (cefazolin 1 g, amoxicillin and clavulanic acid 1.2 g or gentamycin 160 mg) were administered intravenously. A urinary catheter was removed on the morning of the postoperative day. A vaginal gauze pack (gauze soaked in Betadine iodine) was placed for 12 h. The post-voided residual urine was measured by ultrasonography before each patient was discharged. All the patients participated in topical intravaginal oestrogen cream treatment for at least twelve months following the operation (Ovestin® 1 mg/gram daily), but none of the patients took part in preoperative oral hormone replacement therapy. The follow-up period after the modified TVM operation was 36 months.

The historical controls comprised 62 patients with genuine SUI verified by urodynamic examination with grades of POP II–III who had originally undergone a TVM operation (control group I). A further 124 patients with anterior compartment symptomatic POP–Q II and any SUI corrected by anterior *colporrhaphy* operations (control group II) were also operated on by the same two senior surgeons at the departments. The data on the historical controls were retrieved retrospectively from the medical database between January 2008 and December 2009. Eligibility for the operation and consistent collection of outcome measurements (operative characteristics and post-operative findings) were tied to a follow-up schedule which was routinely established in the historical groups. This schedule was followed for the mTVM group as well. The follow-up period in the control groups was also three years. Baseline and follow-up evaluations after six weeks to three years were performed by an experienced urogynaecologist. All the patients were given an appointment for the subsequent medical consultation according to a follow-up arrangement (Table 1). When a patient missed an appointment, a urogynaecologist reminded her by telephone, and, hence, there was no registered loss due to lack of follow-up in our samples. For each case, we matched controls in each control arm (groups I and II) who were as similar as possible in age, systemic diseases, and previous parity and vaginal operations.

**Table 1:** Schedule of assessments/data collection for both

Assessment	Recruitment before intervention phase	Intervention (Surgery)	Follow-up				
			6 weeks	6 months	1 years	2 years	3 years
Written informed consent^a^	x						
Gynaecological examination: incontinence symptoms	x	x	X	x	x	x	X
Gynaecological examination: prolapse	x	x	X	x	x	x	X
Urodynamic examination	x				x		X
Adverse events		x	X	x	x	x	X
Urine culture	x			x	x	x	X

^a^available in the modified transvaginal mesh operated women

The factors studied were the demographic and patient data, the intraoperative findings and postoperative factors. Furthermore, the incidence of systemic chronic diseases that might have a detrimental effect on the healing process (i.e. in *diabetes mellitus* and autoimmune diseases) was also recorded, and displacement of the implant due to chronic coughing in airway diseases was also considered. As concerns the long-term postoperative complications of the sling and mesh procedures, we determined the rejection rate, the presence of de novo urge symptoms (DNUS) or urinary tract infection (UTI), and the need for reoperation. The diagnosis of DNUS was set if detrusor pressure changes were detected in cystometrographic pressures after the surgeries. The postoperative complications that led to reoperation were infection, recurrent descent or incontinence, implant extrusion, chronic pelvic pain and total retention. Prosthetic extrusion was diagnosed by the presence of exposed graft material in the vagina. Post-void residual urine is a measurement of the urine that remains in the bladder less than 20 min following voiding which identifies urinary retention. Bladder volumes that suggest urinary retention are commonly defined as greater than 500 to 600 ml [22, 23]. DNUS was classified as a sudden involuntary contraction of the muscular wall of the bladder causing urinary urgency, an immediate unstoppable urge to urinate with a postoperative onset.

Operative and perioperative complications (six weeks after the procedures) described after mTVM and historical operations were collated, overall frequency within all cases were calculated, and severity was graded using the Clavien–Dindo classification comprising all the follow-up periods. More specifically, additional medication due to deviation from the normal postoperative course (pain, fever, wound infection and minimal bleeding) was categorized as Grade I, UTIs requiring antibiotic treatment and SUI/POP without any postoperative correction, DNUS or blood transfusion were grouped as Grade II, and reoperation performed in general anaesthesia was graded as IIIb [22].

### Statistical analysis

The SPSS 17.0 program package was used to analyse the data. The non-parametric design of the continuous variables was verified with the Shapiro–Wilk test. The categorical and continuous variables were compared with the χ^2^ test and Kruskal–Wallis test, respectively. Univariate logistic regression was employed to determine the odds for continuous variables. Multiple logistic regression was used to adjust the comparisons of the groups in terms of age, previous parity, postmenopausal stage, previous vaginal operations, chronic systemic diseases, POP–Q stage and urge symptoms due to inequalities between cases and controls. A two-tailed *p*-value of < 0.05 was judged as significant. The power of the statistical tests ranged between 74 and 99% in the study.

## Results

Table 2 presents the baseline characteristics of the participants in the study groups. Maternal age and BMI were significantly higher among the mesh-operated patients (*p* < 0.001 and *p* = 0.004, respectively). The vast majority was postmenopausal in all groups; however, the highest rate was noticed disproportionally among those who had undergone mTVM (97%).

**Table 2:** Baseline characteristics of patients who presented for operation for POP–Q II–III and genuine SUI between January 2006 and December 2012

	Modified TVM group (*N* = 62)	Control group I (historical controls: TVM) (N = 62)	Control group II (historical controls: anterior colporrhaphy) (*N* = 124)	*p* value
Age (y) (mean ± S.D.)	62.9 ± 7.7	59.7 ± 10.1	55.9 ± 11.1	< 0.001
BMI (kg/m^2^) (mean ± S.D.)	29.4 ± 2.9	28.4 ± 3.0	27.8 ± 3.8	0.004
Previous vaginal deliveries (mean ± S.D.)	2.3 ± 1.2	2.1 ± 0.6	2.1 ± 0.8	0.71
Postmenopausal women, n (%)	60 (96.8)	47(75.8)	92 (74.2)	0.001
Previous vaginal operations, n (%)	24 (38.7)	14 (22.6)	36 (29.0)	0.14
Chronic systemic diseases, n (%)	17 (27.4)	16 (25.8)	18 (14.5)	0.06
Diabetes mellitus, n (%)	10 (16.1)	10 (16.1)	9 (7.3)	0.09
Autoimmune diseases, n (%)	4 (6.5)	3 (4.8)	5 (4.0)	0.77
Airway diseases, n (%)	5 (8.1)	3 (4.8)	6 (4.8)	0.64

All recruited patients presented for anterior compartment POP–Q II–III (pelvic organ prolapse) and genuine SUI (stress urinary incontinence). The modified transvaginal mesh (mTVM) group comprised patients who received a four–arm mesh that was fixed to the mid-urethra. Control groups I and II include historical controls who had participated in TVM and anterior colporrhaphy without any Kelly–Stoeckel suture, respectively

Chronic systemic diseases include diabetes mellitus, airway diseases and autoimmune diseases

There was a trend of patients with modified mesh having vaginal deliveries and vaginal operations at the highest rate; however, the differences between the groups were not significant. No significant differences were observed between groups in any type of chronic systemic diseases.

Table 3 lists the operative characteristics and complications, while Table 4 provides the odds and adjusted odds of the differences in baseline and surgical characteristics in the three study groups. The operation took a similar amount of time in the mTVM group as compared with the anterior *colporrhaphy* (Adjusted odds rate (AOR): 1.03) or TVM (AOR: 0.97) control group. The estimated blood loss during the operation was significantly lower in the mTVM group than in control group I (AOR: 0.96, *p* < 0.001) and control group II (AOR: 0.96, p < 0.001). The occurrence of bladder injury and the need for immediate postoperative blood transfusion were negligible in all the study groups.

**Table 3:** Operative characteristics and postoperative complications of patients who presented for operation for POP–Q II–III and genuine SUI between January 2006 and December 2012

	Modified TVM group (N = 62)	Control group I (historical controls: TVM) (*N* = 62)	Control group II (historical controls: anterior colporrhaphy) (N = 124)	*p* value
POP grading				
grade II	50 (80.6)	59 (95.2)	124 (100)	< 0.001
grade III	12 (19.4)	3 (4.8)	0 (0)	
Urge symptoms	8 (12.9)	2 (3.2)	7 (5.6)	0.08
Duration of operation (min) (mean ± S.D.)	37.8 ± 7.4	38.8 ± 6.0	34.9 ± 7.9	< 0.001
Estimated blood loss (ml) (mean ± S.D.)	48.7 ± 21.8	83.8 ± 41.2	74.0 ± 33.4	< 0.001
Intraoperative bladder injury, n (%)	1 (1.6)	0 (0)	0 (0)	0.22
Blood transfusion, n (%)	0 (0)	0 (0)	1 (0.8)	0.61
Reoperation, n (%)	4 (6.5)	10 (16.1)	40 (32.3)	< 0.001
POP–Q, n (%)	2 (3.2)	5 (8.1)	31 (25.0)	< 0.001
SUI, n (%)	1 (1.6)	1 (1.6)	11 (8.9)	0.04
Vaginal wall extrusion, n (%)	1 (1.6)	6 (9.7)	0 (0)	0.11
Postoperative bleeding, n (%)	0 (0)	3 (4.8)	1 (0.8)	0.06
Postoperative pain, n (%)	1 (1.6)	0 (0)	0 (0)	0.22
Total retention, n (%)	0 (0)	1 (1.6)	1 (0.8)	0.61
Successful treatment of POP–Q, n (%)	60 (96.8)	56 (90.3)	80 (64.5)	< 0.001
Successful treatment of SUI, n (%)	60 (96.8)	47 (75.8)	76 (61.3)	< 0.001
Urinary tract infection, n (%)	11 (17.7)	14 (22.6)	23 (18.5)	0.75
De novo urge incontinence, n (%)	7 (11.3)	8 (12.9)	0 (0)	< 0.001
Average time to observed extrusion (months) (mean ± S.D.)	1 ± 0	13.7 ± 8.3	n.m.^a^	0.13

All recruited patients presented for anterior compartment POP–Q II–III (pelvic organ prolapse) and genuine SUI (stress urinary incontinence). The modified transvaginal mesh (mTVM) group comprised patients who received a four–arm mesh that was fixed to the mid-urethra. Control groups I and II include historical controls who participated in TVM and anterior colporrhaphy without any Kelly–Stoeckel suture, respectively. n.m.^a^, not measurable

**Table 4:** Crude odds ratios (OR) and adjusted OR (AOR) for confounders in various comparisons of patients who presented for operation for POP–Q II–III and genuine SUI between January 2006 and December 2012

	Modified TVM group vs Control group I (historical controls: TVM)			Modified TVM group vs Control group II (historical controls: anterior colporrhaphy)		
	*p* value	Unadjusted OR (95% CI)	Adjusted OR^a^ (95% CI)	*p* value	Unadjusted OR (95% CI)	Adjusted OR^a^ (95% CI)
Age	0.05	1.04 (1.00–1.08)	1.02 (0.97–1.08)	< 0.001	1.07 (1.04–1.11)	1.05 (1.01–1.10)
BMI	0.05	1.13 (1.00–1.29)	1.12 (0.96–1.30)	0.001	1.14 (1.04–1.25)	1.13 (1.01–1.26)
Previous vaginal deliveries	0.26	1.28 (0.83–1.98)	1.57 (0.90–2.76)	0.45	1.15 (0.84–1.58)	1.18 (0.79–1.76)
Postmenopausal women	0.001	9.6 (2.09–4.35)	11.58 (1.78–75.1)	< 0.001	10.44 (2.41–45.2)	8.09 (0.98–67.05)
Previous vaginal operations	0.08	2.16 (0.99–4.74)	2.16 (0.86–5.44)	0.19	1.54 (0.81–2.93)	1.29 (0.59–2.80)
Chronic systemic diseases	1.00	1.09 (0.49–1.41)	0.95 (0.37–2.41)	0.045	2.23 (1.05–4.71)	1.84 (0.77–4.41)
Diabetes mellitus	1.00	1.00 (0.38–2.60)	0.77 (0.16–4.46)	0.07	2.46 (0.94–6.11)	0.86 (0.19–3.88)
Autoimmune diseases	1.00	1.36 (0.29–6.33)	3.23 (0.41–25.67)	0.48	1.64 (0.43–0.34)	1.99 (0.34–11.59)
Airway disorders	0.72	1.72 (0.40–2.58)	0.90 (0.15–5.54)	0.51	1.73 (0.51–5.89)	0.4 (0.08–2.14)
POP–Q III vs II	0.02	4.71 (1.26–17.5)	6.32 (1.07–37.52)	< 0.001	1.24 (1.10–4.40)	1.36 (1.08–4.10)
Urge symptoms	0.09	4.44 (0.90–21.74)	4.85 (0.91–25.94)	0.10	2.48 (0.85–7.18)	3.02 (0.84–10.90)
Duration of operation	0.41	0.98 (0.93–1.03)	0.97 (0.91–1.03)	0.22	1.05 (1.01–1.09)	1.03 (0.98–1.09)
Estimated average blood loss	< 0.001	0.96 (0.95–0.98)	0.96 (0.94–0.98)	< 0.001	0.96 (0.94–0.97)	0.96 (0.94–0.98)
Intraoperative bladder injury^b^	1.00	1.02 (0.98–1.05)	n.m.^b^	0.33	1.02 (0.99–1.05)	n.m.^b^
Blood transfusion	n.m^b^	n.m^b^	n.m.^b^	1.00	0.99 (0.98–1.01)	n.m.^b^
Reoperation^b^	0.15	0.36 (0.11–1.21)	0.22 (0.04–1.10)	< 0.001	0.15 (005–0.43)	0.07 (0.02–0.32)
POP	0.44	0.38 (0.07–2.04)	0.26 (0.04–1.96)	< 0.001	0.10 (0.02–0.43)	0.05 (0.006–0.41)
SUI	1.00	1.00 (0.06–16.39)	0.19 (0.01–5.7)	0.06	0.17 (0.02–1.34)	0.11 (0.06–0.89)
Vaginal wall extrusion	0.11	0.15 (0.02–1.31)	0.13 (0.009–1.71)	n.m	n.m.^b^	n.m.^b^
Bleeding	0.24	0.95 (0.90–1.01)	n.m.^b^	1.00	0.99 (0.98–1.01)	0.89 (0.68–1.12)
Postoperative pain	1.00	1.02 (0.98–1.05)	n.m.^b^	0.33	1.02 (0.99–1.05)	n.m.^b^
Total retention	1.00	0.98 (0.95–1.02)	n.m.^b^	1.00	0.99 (0.98–1.01)	n.m.^b^
Successful treatment of POP–Q	0.27	3.22 (0.62–16.67)	2.94 (0.47–18.6)	< 0001	16.5 (3.85–70.77)	14.16 (3.09–65.00)
Successful treatment of SUI	0.004	9.58 (2.09–43.9)	72.2 (8.56–606.65)	< 0.001	18.95 (4.43–81.13)	96.2 (7.40–1250.0)
Urinary tract infection	0.66	0.74 (0.31–1.79)	0.70 (0.26–1.87)	1.00	1.95 (0.43–2.19)	0.92 (0.34–2.45)
De novo urge symptoms	1.00	0.86 (0.29–2.53)	0.89 (0.25–3.22)	< 0.001	1.13 (1.03–1.23)	1.10 (1.06–1.19)
Average time to extrusion observed	0.99	n.m.^b^	n.m.^b^	n.m.^b^	n.m.^b^	n.m.^b^

All recruited patients presented for anterior compartment POP–Q II–III (pelvic organ prolapse) and genuine SUI (stress urinary incontinence). The modified transvaginal mesh (mTVM) group comprised patients who received a four–arm mesh that was fixed to the mid-urethra. Control groups I and II include historical controls who participated in TVM and anterior colporrhaphy without any Kelly–Stoeckel suture, respectively

OR, Odds ratio; 95% CI, 95% confidence interval,

Adjusted OR^a^, All variables were adjusted for age, previous parity, postmenopausal stage, previous vaginal operations and chronic systemic diseases, POP–Q stage and urge symptoms. n.m.^b^, not measurable

The reoperation rate was significantly the lowest in the mTVM group at 6.5% as compared to that in the anterior *colporrhaphy* group (32.1%) (AOR: 0.07, p < 0.001) and that of 16.1% in the TVM group (AOR: 0.22, *p* = 0.15). The rate of implant removal was lower among those mesh-operated patients who had undergone the modified technique than those with the original TVM (AOR: 0.13, 1.6% vs 9.7%, respectively); however, the difference did not reach a significant level. One reoperation occurred in the mTVM group due to apical compartment POP (1.6%), another (1.6%) was performed due to anterior compartment descent after the removal of the extruded mesh, a third (1.6%) for SUI and a fourth (1.6%) for postoperative pain. In addition, reoperation due to postoperative POP had a frequency of 8.1%, and one patient (1.6%) required reoperation due to SUI in the original TVM group. The *colporrhaphy* patients needed more reoperations (32.1%), and the difference was robust (AOR: 0.007). All the cases involving POP recurrence (3.2 and 9.7%, AOR: 2.94) following the two mesh operations were found in the untreated compartment, posteriorly behind the mesh-supported area. The recurrence of anterior compartment POP (35.5%, *p* < 0.001) and SUI (38.7%, *p* < 0.001) or reoperation due to recurrence of SUI (8.9%, AOR: 0.11, *p* = 0.06) and POP (25.0%, AOR: 0.05, p < 0.001) during the follow-up period was typical of the anterior *colporrhaphy* patients. As expected, slightly more cases of SUI were cured in the mTVM group than in the TVM group (96.8% vs 91.9%), although the differences were not significant (AOR: 2.98). Prolapse repair was achieved in a significantly higher proportion of the patients who underwent mTVM compared to their anterior *colporrhaphy* counterparts (96.8% vs 64.5%, AOR: 14.16 p < 0.001). Modification of the TVM displayed a moderate effect on POP recurrence compared to the original operation (9.7%) (AOR: 2.94, *p* = 0.27). Urinary tract infection was not more prevalent after the prosthesis operations than after anterior *colporrhaphy*. The rate of extrusion was nearly four times higher in the TVM group than in the mTVM group (AOR: 0.13, *p* = 0.11), with extrusion appearing earlier in the group that underwent a non-modified operation. All implants were removed because of the extrusion of the vaginal wall. No rectal or bladder *fistula*, pelvic abscess or *haematoma* was observed in any of the groups.

Modifying the TVM technique led to a signicantly higher elevation of point Aa compared to the traditional TVM (AOR: 4.83) with a significantly reduced shortening of the vagina (AOR: 0.41). The mTVM significantly improved the prolapse status (POP–Q of Aa, AOR: 142.9; Ba, AOR: 5.95; and C, AOR: 25.0) compared to that of anterior colporrhaphy, whereas the total vaginal length was significantly shortened (*p* < 0.001) (Table 5).

**Table 5:** Patients’ preoperative and postoperative POP-Q status

	Control group I (historical controls: TVM) (N = 62)			*p* value	Unadjasted OR (95% CI)	Adjusted OR^a^ (95% CI)	Modified TVM group (*N* = 62)			*p* value	Unadjasted OR (95% CI)	Adjusted OR^a^ (95% CI)	Control group II (historical controls: anterior colporrhaphy) (*N* = 124)		
	Pre-operative	Post-operative	Mean difference				Pre-operative	Post-operative	Mean difference				Pre-operative	Post-operative	Mean difference
Aa	−0.97 ± 0.6	−1.37 ± 0.55	−0.40 ± 0.61	< 0.001	4.55(2.38–9.09)	4.83 (2.26–10.31)0.6	− 0.55 ± 0.69	− 1.68 ± 0.74	− 1.13 ± 0.80	< 0.001	43.5(1.61–125)	142.9(28.57–500.0)	−0.97 ± 0.36	− 0.76 ± 0.69	0.21 ± 0.60
Ba	−0.42 ± 0.64	− 1.74 ± 1.0	−2.16 ± 1.04	0.50	1.12 (0.8–1.56)	1.17(0.71–1.52)	−0.53 ± 1.08	−1.76 ± 0.94	−2.3 ± 1.08	< 0.001	6.49(3.65–11.63)	5.95(3.27–10.87)	− 0.49 ± 0.75	− 0.94 ± 1.48	− 0.45 ± 1.17
C	−6.55 ± 1.16	−6.82 ± 0.85	−0.27 ± 0.6	0.70	0.90 (0.54–1.52)	1.20(0.66–2.16)	− 6.53 ± 0.82	− 6.76 ± 0.67	− 0.23 ± 0.58	< 0.001	23.81(3.34–166.67)	25.0(3.70–166.7)	− 6.74 ± 0.61	− 6.45 ± 0.83	0.29 ± 0.62
TVL	8.25 ± 0.67	7.9 ± 0.62	−0.35 ± 0.48	0.018	0.37(0.16–0.86)	0.41(0.16–0.99)	7.82 ± 0.42	7.66 ± 0.60	−0.16 ± 0.41	< 0.001	n.m.^b^	n.m.^b^	7.91 ± 0.29	7.91 ± 0.29	0.0 ± 0.0

All recruited patients presented for anterior compartment POP–Q II–III (pelvic organ prolapse) and genuine SUI (stress urinary incontinence). The modified transvaginal mesh (mTVM) group comprised patients who received a four–arm mesh that was fixed to the mid-urethra. Control groups I and II include historical controls who participated in TVM and anterior colporrhaphy without any Kelly–Stoeckel suture, respectively

OR, Odds ratio; 95% CI, 95% confidence interval,

Adjusted OR^a^, All variables were adjusted for age, previous parity, postmenopausal stage, previous vaginal operations and chronic systemic diseases, POP–Q stage and urge symptoms. n.m.^b^, not measurable

Table 6 demonstrates the postoperative complications within six weeks according to the Clavien–Dindo classification. A total complication rate of 11.3% in the mTVM group, 35.5% in the TVM group and 22.6% in the *colporrhaphy* group was noted with significant differences (*p* = 0.013). Subanalyses were performed indicating that the complications occurred with a non-significantly lower prevalence among the mTVM participants compared to the anterior *colporrhaphy* group (AOR: 0.56, *p* = 0.3), whereas the difference was significantly lower in the mTVM group than among the TVM participants (AOR: 0.29, *p* = 0.02) (data are not shown). CD I complications predominantly occurred in the two groups of women operated on with prostheses, whereas the historical anterior *colporrhaphy* operations were followed mostly by CD II.

**Table 6:** Clavien–Dindo classification for postoperative surgical complications among patients who presented for operation for POP–Q II–III and genuine SUI between January 2006 and December 2012

Clavien–Dindo grade^a^	Complications	Modified TVM group (*N* = 62)	Control group I (historical controls: TVM) (*N* = 62)	Control group II (historical controls: anterior colporrhaphy) (*N* = 124)	*p* value
I	Non–pharmacologically treated postoperative bleeding, fever, wound infection	5 (8.1)	12 (19.4)	11 (8.9)	0.013
II	Urinary tract infection, post–void residual volume, blood transfusion, treatment for de novo urge symptoms	1 (1.6)	7 (11.3)	16 (12.9)	
IIIb	Reoperation for vaginal wall extrusion	1 (1.6)	3 (4.8)	1 (0.8)	
Total complications		7 (11.3)	22 (35.5%)	28 (22.6)	0.015

CD, Clavien–Dindo. ^a^No grade IIIa, IVa, IVb or V complication occurred. ^b^Data are expressed as n (%)

## Discussion

The most striking result of this study is that the transvaginal implantation of the four-arm mesh sutured to the mid-*urethra*, a new modification of the TVM procedure [7], is highly effective in the repair of an *anterior* prolapse (POP–Q II–III) and in genuine stress urinary incontinence (SUI). Success rates of 96.8 and 96.8% were demonstrated for genuine SUI and prolapse, respectively. This surgical procedure for prosthetic placement provided a minimally better SUI reconstructive effect than that of the TVM procedure in our historical control group (91.9%) or that reported in the literature (69–90.3%) [24–26]. With regard to the POP repair, mTVM (96.8%) proved significantly more effective than historical anterior colporrhaphy (64.5%), but only minimally higher than that of the non-modified, transobturator mesh technique in our database (90.3%) and that demonstrated in the literature (with a rate of 82.3–100%) [5, 20, 21, 26–29]. Fixing the *anterior* arm of the mesh to the *periurethral* tissue elevates point Aa significantly more effectively than the original TVM with less vaginal shortening.

TVM supports the whole of the anterior and middle compartments, but does not elevate the middle of the *urethra* and, hence, theoretically allows for urethral dorsal rotation, which may lead to residual SUI, especially if the mesh is able to migrate a bit more posteriorly toward the cervix. It is also possible that mesh movements could be responsible for DNUS with the traditional TVM method. Using stabilizing sutures to fix the mesh to the paraurethral tissues at the level of the mid-*urethra*, where the *pubourethral* ligament originally held it, helps to elevate the middle region, this being considered the treatment of SUI that is at least as effective as the TVT-O technique (91.2% in our non-published data and 92% in the literature) [30]. In other words, anchoring the anterior part of the mesh to the mid-*urethra* imitates the mechanism of action of the mid-*urethral* sling as an additional procedure for SUI correction. The original TVM has a limited anti-SUI mechanism.

Furthermore, our results suggest a better anatomical success rate after the mesh is fixed to the mid-*urethra*, which may be explained by the lack of shrinkage and less folding of the mesh. By contrast, our results might allow for the speculation that prosthesis stabilization with sutures to prevent any backward movement could lead to prolapse recurrence in the untreated middle compartment, i.e. in the area not supported by the mesh.

We attribute our lower anatomical recurrence of mTVM to a wider suspension area of the insertion of a smooth, non-folded mesh. To check on this, we have launched a further study at our department, in which the posterior arms of the TVM are anchored towards the pericervical ring to provide apical support.

TVM corrects SUI subjectively in only 83.3% of cases [7]. Furthermore, subvesical mesh towards the obturator spaces in a total of 74 patients with a stage ≥2 anterior prolapse was associated with a symptomatic SUI repair in 72% of the cases, and cystocele was corrected in 97% [27]. The current trend is to resolve the concomitant SUI and POP in a single hospitalization session so as to reduce medical costs and improve the patient’s satisfaction, but the optimum use of anti-incontinence procedures during pelvic reconstructive surgery remains a matter of debate [31]. A combination of mesh with a mid*-urethral* sling in one session has been scarcely studied [12, 32]. When TVM was combined with a TVT-O operation [12, 32], the success rate for SUI was 87.8–93.2% and that for anterior wall repositioning was 94.4–96%, i.e. a similar anti-incontinence and anatomical reconstruction rate than those in our study, though that follow-up was for up to 60 months. More studies are needed to evaluate whether the additional insertion of a TVT-O sling into a mesh provides any additional improvement in the SUI and POP cure rate as compared with a single TVM or modified TVM.

Surprisingly, the double-sling extrusion rate was extremely low (0–10%) [12, 32]. The corresponding figures were 1.6% in our mTVM group and 9.7% among our TVM participants, which is in agreement with the mean value of 11.6% (ranging from 3.9 to 16%) reported in the literature [4, 20, 24, 27, 33, 34]. The extrusion rate generally correlates well with the prosthesis breadth; however, we observed a remarkably higher extrusion rate for mesh compared to that for modified mesh. One can speculate that the lack of stabilizing sutures led to a “folding/wrinkling” of the edge of the mesh, resulting in a lifting up and compressing of the mucosa and deranged periprosthetic vasculature. The reported incidence of prosthesis exposure for double-sling operations is not clear-cut. A wider and double prosthesis involves a greater chance of bacterial colonization, and the enhanced preparation may additionally impair blood supply to the vaginal wall.

Anterior *colporrhaphy* is inferior to mesh procedures with regard to the effectiveness of POP repair and anti-incontinence both in our database and as reported by others (37–85 and 54%, respectively) [20, 33, 35, 36]. The mTVM greatly improved the POP–Q status in all examined compartments in the vagina compared to that of colporrhaphy. However, the perioperative complication rate is diminished after non-mesh surgery (22.6%) compared to that after implant surgery (35.5%). This is supported by the literature data [20, 28, 35]; however, our modified mesh procedure produced the lowest complication rate in our dataset (11.3%). The robustly lower complication rate after the mTVM can be partly explained by the fact that the surgeons in the study received more training at the time. It is of note that there are no studies which illustrate the perioperative, short-term complications of these genitourinary surgeries.

In a single TVM series with the Perigee technique, the average blood loss was found to be 180 ml [33], which was much higher than in our mTVM data series (48.7 ml), and the mean procedure duration was also shorter in the mTVM group (37.8 min vs. 60.9 min) [36].

One of the most frequent long-term complications we observed with synthetic mesh materials in our series was DNUS (11.3% and 12.9), which is an important indicator of the level of patient satisfaction. Post-operative urodynamic examinations showed detrusor muscle action in all the cases suffering from DNUS. The average detrusor muscle pressure was 7.75 cmH_2_O postoperatively, whereas no sign of detrusor or intravesical pressure elevation was detected preoperatively. We believe that one possible reason for this is the fact that the mesh cannot slide backwards and cannot press the *urethra* to the bladder neck after the TVM is modified. The average reported prevalence of de novo dyspareunia following vaginal placement of a polypropylene mesh is 14% [37], i.e. significantly higher than in our study (1.6%) (the data are not presented in a table). Mesh+sling operations impair the pelvic blood circulation and induce de novo urge symptoms and pelvic pain; however, the reported extrusion rate was 10% [32].

Our study had some limitations. The baseline characteristics of the patients were not equally distributed in the groups, and, hence, statistical adjustments were used in multivariate analyses. A further limitation of the study is its non-randomized manner. The surgeons became better trained in the operative techniques, a fact which may somehow bias the results. Moreover, it was not an aim of the study to determine the subjective curative rate of SUI or POP; however, the prospectively collected objective curative frequency was noted. Furthermore, different types of complications occur after anterior *colporrhaphy* compared to mesh operations (i.e. extrusion does not occur after *colporrhaphy*), and the total complication rate should be interpreted with caution.

## Conclusions

In summary, use of a modified transvaginal mesh resulted in a higher rate of treatment success than the traditional mesh operation or non-mesh reconstructive surgery (*colporrhaphy)* for repair of anterior vaginal-wall prolapse and stress incontinence. By seeking a balance between the highly effective anti-incontinence and POP repair of the prosthesis operations and the less harmful non-mesh repairs, the mTVM could be a reasonable choice with a high SUI and coexisting POP reconstructive effect, thus ensuring lower rates of intra- and postoperative adverse events. Further randomized studies are therefore necessary to compare the efficacy of this novel intervention with that of double-sling procedures.

## Abbreviations:

CD: Clavien–Dindo classification system
DNUS: De novo urge symptoms
ISD: Intrinsic sphincter deficiency
mTVM: Modified transvaginal mesh operation
PFMT: Pelvic floor muscle training
POP: Pelvic organ prolapse
POP–Q II: Pelvic organ prolapse quantification system stage II
SUI: Stress urinary incontinence
TVL: Lotal vaginal length
TVM: Transvaginal mesh operation
TVT-O: Transobturator tape operation
UTI: Urinary tract infection
